# Effectiveness of school-based psychological interventions for the treatment of depression, anxiety and post-traumatic stress disorder among adolescents in sub-Saharan Africa: A systematic review of randomized controlled trials

**DOI:** 10.1371/journal.pone.0293988

**Published:** 2023-11-20

**Authors:** Minale Tareke, Biksegn Asrat Yirdaw, Abebaw Gebeyehu, Bizu Gelaye, Telake Azale

**Affiliations:** 1 Psychiatry Department, College of Medicine and Health Science, Bahir Dar University, Bahir Dar, Ethiopia; 2 Department of Psychiatry, College of Medicine and Health Sciences, University of Gondar, Gondar, Ethiopia; 3 UK Public Health Rapid Support Team, UK Health Security Agency/London School of Hygiene and Tropical Medicine, London, United Kingdom; 4 Centre for Global Mental Health, London School of Hygiene and Tropical Medicine, London, United Kingdom; 5 JSI-Data Use Partnership, Ministry of Health, Addis Ababa, Ethiopia; 6 Department of Psychiatry, MD Division of Global Psychiatry, Harvard T. H. Chan School of Public Health, Harvard Medical School and The Chester M. Pierce, Massachusetts General Hospital, Boston, Massachusetts, United States of America; 7 Institute of Public Health, College of Medicine and Health Sciences, University of Gondar, Gondar, Ethiopia; Medical University of Vienna, AUSTRIA

## Abstract

**Background:**

Mental disorders among adolescents represent a high burden and early onset. They compromise their physical health, survival, and future potential. On the other hand, young people have inadequate access to essential health services in sub-Saharan Africa. We aimed to review school-based psychological interventions, contents, delivery, and evidence of effectiveness designed to treat depression, anxiety, or posttraumatic stress symptoms among adolescents and young adults aged 10–24.

**Method:**

We searched articles on the following databases: PubMed, Scopus, Embase, and Science Direct from 17/10/2022 to 30/12/2022. Furthermore, relevant studies were searched from advanced google scholar, google and identified reference lists. We used MeSH browser for key words: psychological interventions, depression, anxiety, posttraumatic stress disorder and lists of Sub-Saharan Africa countries. We combined words using standard Boolean operators (OR, AND). The quality of studies was evaluated using the Cochrane Collaboration’s risk of bias tool and the results were presented as a narrative synthesis since the interventions were very heterogenous.

**Results:**

Fourteen randomized controlled trials were included for systematic review and more than half (57.14%) were from Kenya and Nigeria. Common school-based psychological interventions were cognitive behavioral therapy and Shamiri interventions (an intervention that focuses on youths to cultivate a growth mindset, practice gratitude and take the value). More than half (57.14%) of the interventions were delivered by non-specialists like teachers, lay providers and community health workers. Nearly one-fifth of the interventions were used individual modality. School-based psychological interventions provided by non-specialists also produced a greater reduction in adolescents’ depressive, anxiety, and post-traumatic stress symptoms compared to the control groups.

**Conclusion:**

Cognitive behavioral therapy and Shamiri interventions were the common treatment delivered in school settings. The range of interventions could be effectively delivered by non- professionals that promote task-shifting of psychological interventions from very scarce mental health specialists in these countries.

**Trial registration:**

**Trial Registration: Prospero** CRD42022378372. https://www.crd.york.ac.uk/prospero/display_record.php?ID=CRD42022378372.

## Introduction

Mental disorders are responsible for a large proportion of burden of disease among adolescents in all societies. Most mental health conditions begin early in life (12–24 years of age): 50% arising before age 14 and 75% by 24, even though they are often first identified later in life [1]. In 2019, one adolescent from seven (166 million adolescents) experienced mental health problems, 90% living in low- and middle-income countries (LMICs) [2].

According to the Lancet Child and Adolescent Health published in 2018, the definition of adolescent age is more expanded and inclusive from 10–19 years to 10–24 years. This is the delayed role transitions that include completion of education, marriage, and parenthood shifts familiar perceptions of when adulthood begins. The age of 10–24 years more closely to adolescent growth and understandings of the life phases and facilitate extended investments across a wide range of settings. It is essential for developmentally appropriate framing of laws, social policies, and service systems [3].

The Sub-Saharan Africa (SSA) has the highest rate of disability-adjusted life years and the fastest-growing adolescent population in the world [4]. However, the mental health treatment gap is inefficient, inadequate, and inequitable. Most adolescents in SSA are left with no alternative except to suffer with untreated mental illnesses or seek help for psychological issues from traditional or religious leaders [5].

Mental disorders among adolescents represent the main threat to their health, survival, and future potential worldwide [6]. The most prevalent mental health conditions in young people are depression, anxiety disorders, posttraumatic stress disorder, and suicidal behavior [7] with detrimental impacts on their survival, growth, and development. They have also been linked to HIV/AIDS, other STDs, domestic violence, teenage pregnancies, car accidents, physical fights, crime and homicide [8].

Despite the high burden and impacts, most mental health conditions remain unrecognized and untreated [9]. Many African countries do not have adequate, functioning, and resilient public health systems [10]. Adolescents have the worst access to timely and quality mental health care [6]. For example, in Sierra Leone, the treatment gap for formal child and adolescent mental health services is estimated at 98·8% [11]. The issue is compounded by a significant shortage of specialized mental health practitioners including psychiatrists and psychiatric nurses [12]. To alleviate these challenges, WHO launched in 2010 Mental Health Gap Action Program Intervention Guide (mhGAP-IG) for non-specialists to give mental health services with integration into existing systems in LMICs [13]. However, for the implementation and sustainability of mhGAP, cultural factors identified major barriers to improve services [14].

Intervention development and adaptation have been primarily focused on HIV/AIDS service delivery in school or community programs by non-specialist health workers. There is a critical need for child and adolescent mental specialist, integration of those services into primary healthcare, coordinated cross-sectoral cooperation, and established referral networks [15]. Evidence showed that school-based psychological interventions are effective in depressive symptoms reduction, anxiety, suicide, substance use, aggression, and violence [16–18]. Network meta-analysis compared psychological therapy with antidepressants for the management of depression and found comparable effects on remission, dropouts, and depression symptoms [16,19,20].

Psychological interventions in school enhance academic achievement by addressing self-esteem and social well-being [21]. Adolescents with mental health problems are more vulnerable to academic failure, dropping out of school, substance abuse, criminal/legal involvement, and exploitation [21]. Schools play an essential role in supporting many young people’s mental health and can also enable early intervention when displaying symptoms [22]. The school setting also provides an opportunity to reach large numbers of young people simultaneously [23].

There are trials of school-based psychological interventions carried out in SSA countries among adolescents with the need for summarized relevant studies to provide a better treatment appropriate to the culture. The studies were too heterogenous to pooled using meta-analysis in terms of psychological intervention in principles, duration of therapy, session duration, delivery mode (individual or group), content, different approaches, and outcome measurements. One of the recommendations by Cochrane Handbook, the results will not be pooled using meta-analysis if there are substantial heterogeneity found because it may produce misleading results [24]. Therefore, we synthesize a narrative of quantitative studies without meta-analysis.

This systematic review focused only on randomized controlled trials (RCTs) since they have a lower risk of bias compared to other study designs. Therefore, the findings from this review provide the basis for practitioners working to improve the mental health of adolescents, policy planners, and researchers involved in developing and delivering school-based mental health initiatives.

The review aimed to assess school-based psychological interventions’ contents, delivery and evidence of effectiveness designed to treat depression, anxiety, or PTSD among adolescents aged 10–24.

We used the PICO framework for the systematic review question: are school-based psychological interventions (intervention) effective for adolescents (population) compared to usual or other care (comparison) on depressive, anxiety, or post-traumatic stress symptoms (outcome) in SSA)?

## Methods

We used the Cochrane Handbook for the systematic review of interventions version 6.3 and reported according to the Preferred Reporting Items for Systematic Reviews and Meta-analysis (PRISMA) 2020 guidelines [25] (See S1 Checklist). We used Cochrane Handbook because it provides detailed methods on systematic reviews on the effect of an intervention. It was prospectively registered in PROSPERO (CRD42022378372) [26].

### Inclusion criteria

Articles eligible for inclusion were required to demonstrate the following characteristics:

1. **Population**: participants were adolescents (10–24 years). This is a broader and more encompassing definition of adolescence to frame laws, social policies, and service systems in a manner that is developmentally appropriate [3].
2. **Intervention:** school-based psychological interventions conducted in SSA and designed to reduce depressive, anxiety and PTSD symptoms
3. **Comparison**: control group that include no intervention, usual care, alternative intervention, or waitlist
4. **Outcome**: The psychological treatment outcomes included depression, anxiety, or post-traumatic stress disorder (PTSD)
5. **Study design:** Randomized controlled trials conducted in SSA. The restriction of SSA intended to narrow down the scope of review to countries that share similar socio-economic, cultural, and political situations.
6. **Context:** Published in English language (due to limitation of others language by the team) from 2010–2022. The year 2010 was also selected because WHO launched the Mental Health Gap Action Program (mhGAP) to scale up care and services using evidence-based interventions for preventing and managing mental illness, including child and adolescents mental health problems [27].

There were exclusion criteria:

1. Health institutions-based interventions
2. Studies that did not measure depression, anxiety or PTSD as an outcome
3. Studies that included adults and adolescents without presenting results separately by age
4. Studies evaluated mixed interventions, such as both psychological and pharmacological interventions.
5. Trials of interventions delivered in universities or colleges. We believed that all college/university students in SSA not under the age of 24 years and the nature of stressors somewhat different from secondary school students.

### Search strategy

In the beginning, index terms and keywords were identified based on the participants, intervention, comparator and outcome (PICO) framework [28]. We systematically searched the following electronic databases: PubMed, Scopus, Embase, and ScienceDirect from 17/10/2022 to 30/12/2022. Additionally, we searched gray literature from advanced Google scholar and google in the same dates. We restricted the search strategy to the English language, randomized controlled study design and publication date from 2010–2022.

We used MeSH browser for key words: psychological interventions, depression, anxiety, posttraumatic stress disorder and lists of Sub-Saharan Africa countries. We combined words using standard Boolean operators (OR, AND). The key terms used to retrieve primary articles were adolescent* OR youth* OR teen* OR “young people” AND depression OR “depressive symptoms” OR “depressive disorder” OR “affective disorder” OR “major depression” OR “major depressive disorder” OR “probable depression” OR “anxiety disorders” OR “social anxiety” OR anxiousness OR anxious OR anxiety OR “internalized problems” OR “emotional problems” OR “post-traumatic” OR posttraumatic OR PTSD OR “psychological distress” AND “group psychological interventions” OR “brief psychosocial interventions” OR “psychosocial interventions” OR “psychological interventions” OR “psychological treatment” OR “psychological therapy” OR “psycho-supportive interventions” OR psychoeducation OR “self-help intervention” OR “stress management” OR “emotional support” OR “interpersonal therapy” OR “behavioral intervention” OR “behavioural activations” OR “cognitive behavioural therapy” OR “cognitive behavioral therapy” OR “problem solving” OR “group therapy” OR “social skill interventions” OR psychotherapy OR counseling OR mindfulness AND lists of Sub-Saharan Africa countries (see S1 File).

### Study selection

We removed duplicate articles using endnote (version 20.0.0.14672) before starting the two-stage selection process. Two independent authors (MT and BA) read study titles and abstracts based on eligibility criteria and classified references: yes (included), maybe (included) and no(excluded). Any disagreement was resolved via consensus or by involving a third author (TW). Abstracts in which eligibility was unclear underwent full-text review. Then, the remaining full-text articles were assessed for inclusion by two authors (MT and BA). Furthermore, relevant studies were searched from identified reference lists manually.

## Quality assessment

Two reviewers critically appraised the internal validity of eligible studies using the Cochrane Collaboration’s risk of bias tool (RoB 2) [29]. The RoB 2 tool is structured in the following domains for risk of bias: randomization process, deviations from intended interventions, missing outcome data, measurement of the outcome and selection of the reported result. Then, the two reviewers (MT, BA) independently judged the quality assessment using the criteria: high, low, or unclear risk). Any disagreements were resolved with the discussion between the two reviewers, if not by involving the third authors. Based on the appraisal of each criterion, an overall risk of bias assessment was made: high, moderate, low, and unclear. A quantitative cut-off score was not used to determine the methodological quality. However, only studies with a low or moderate overall risk of bias were included in the summary of evidence. Studies were considered a low risk of bias when all-important contents were judged and found to be at low risk. Trials with a high risk of bias in at least one domain were rated as having an increased risk of bias [30]. The kappa coefficient (inter-agreement between the two raters) was found to be 0.76.

## Data extraction

We used data extracted based on recommendations by the Cochrane Handbook for Systematic Reviews of Interventions. Two reviewers (MT and BA) extracted data independently using a data extraction form that includes: the name of the first author, year of publication, country of study, age of participants, sample size, type of intervention, number of sessions, length, comparison group and study outcomes, the effect of the interventions. Data were extracted into a Microsoft Excel spreadsheet (See S2 File).

## Result

### Search results

**Fig 1:**
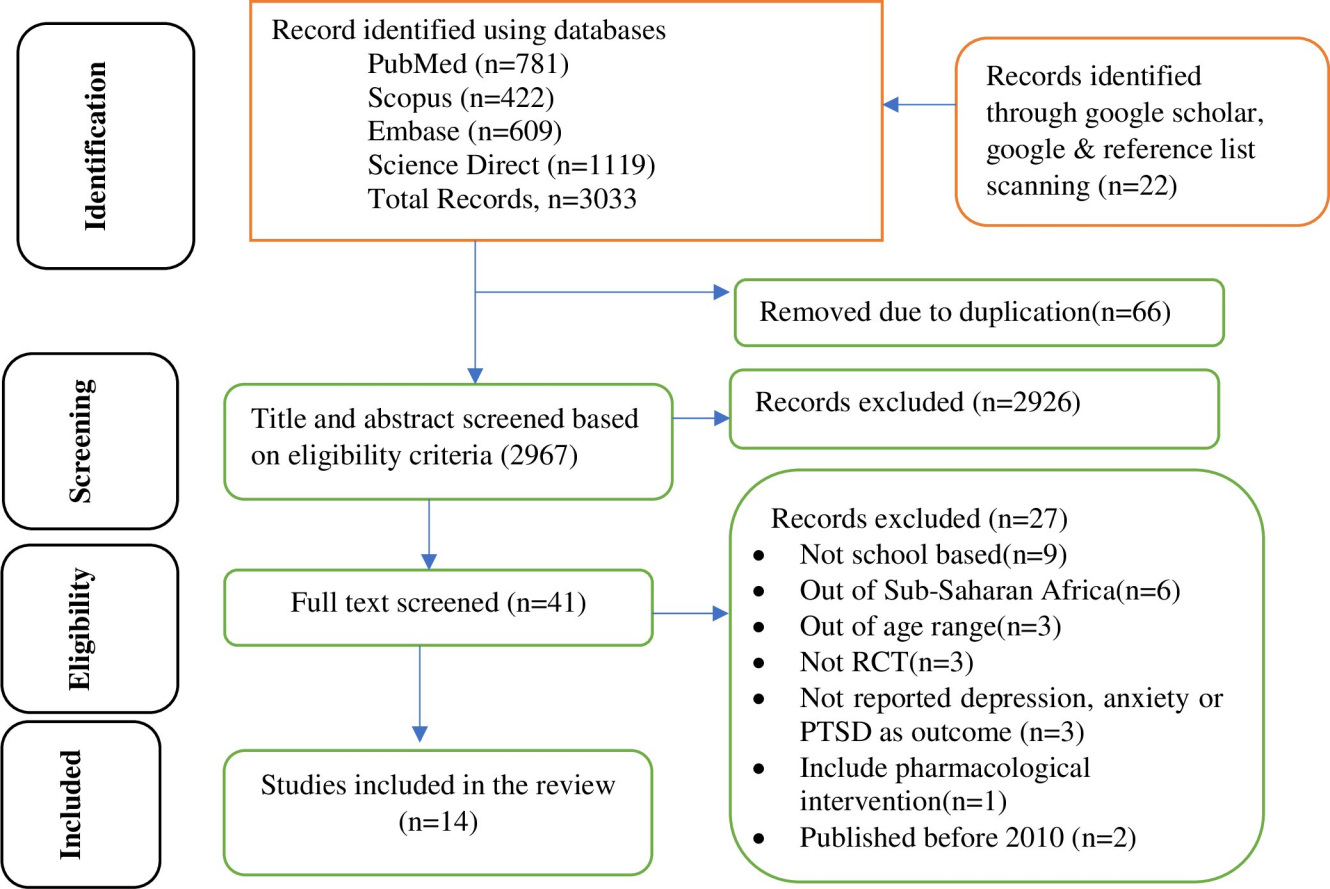
PRISMA 2020 flow diagram.

Search results were summarized in the PRISMA 2020 flow diagram. Out of 3033 articles retrieved, 66 articles were removed due to duplication through EndNote 20 citation manager. Then, 2967 papers were excluded after the title and abstract screening based on inclusion criteria. The remaining forty-one full texts were checked in detail for inclusion criteria again and only fourteen RCTs were included for systematic review. The main reasons for twenty-seven full text exclusions were: not school-based (community and health facilities) studies, out of SSA, out of age range, not randomized controlled trials, including pharmacological intervention and not reported depression, anxiety, or PTSD as an outcome (Fig 1).

### Quality assessment

From the included RCTs, only three were found to have a low risk of bias [31–33]. The remaining met some concerns [34–41] and a high risk of bias [42–44]. Six RCTs out of the fourteen did not provide their trial registration numbers [36,39,40,42–44] (Table 1).

**Table 1:** Cochrane risk of bias assessment for included studies (RoB 2 tool) (n = 14).

	Study (author and year)	D1	D2	D3	D4	D5	Overall
1	Rossouw -2018 [34]	+	**-**	+	+	**-**	**-**
2	Bella‐Awusah-2016 [42]	+	x	-	-	-	x
3	Thurman-2017 [32]	+	+	+	+	+	+
4	Betancourt-2014 [35]	+	+	-	+	-	-
5	Unterhitzenberger-2014 [43]	+	x	+	-	-	x
6	Jibunoh-2022 [44]	-	x	+	-	-	x
7	Rivet‐Duval-2011 [36]	-	-	+	+	+	-
8	Getanda-2020 [37]	+	-	+	+	+	-
9	Osborn-2021 [31]	+	+	+	+	+	+
10	Osborn-2020 [38]	+	-	+	+	+	-
11	Osborn-2022 [39]	+	-	+	+	+	-
12	Are-2022 [40]	-	-	+	+	+	-
13	Byansi-2022 [41]	+	-	+	+	-	-
14	Venturo-Conerly 2022 [33]	+	+	+	+	+	+

Key: Color representation as follows: Green (low risk), red (high risk) and yellow (some concerns).

D1: Randomization process, D2: Deviations from intended interventions (effect of assignment to intervention or effect of adhering to intervention).

D3: Missing outcome data, D4: Measurement of the outcome, D5: Selection of the reported result and D6: Overall risk of bias.

### Psychological interventions characteristics

Overall, 4262 individuals were represented from seven countries in SSA. From fourteen included studies, the highest number reported from Kenya (four) [31,33,37–39], Nigeria (three) [40,42,44] and South Africa (two) [32,34]. The remaining studies where from Sierra Leone [35], Mauritius [36], Rwanda [43] and Uganda [41]. Over three fourth of the studies (78.6%, 11/14) were published after 2016. The sample size varied wildly from 40 participants [44] to 1260 participants [41]. The mean age of the study respondents also approximately 15.5 years old.

The details of intervention characteristics include content, mode of delivery, duration, number of sessions, who delivered the intervention, and the control group found in the Table 1. The types of school-based psychological interventions include Shamiri interventions (n = 3) (an intervention that focuses on youths to cultivate a growth mindset, practice gratitude and take the value or virtues) [31,33,38], cognitive behavioral technique (n = 4) [35,36,40,42], psycho-social-educational group intervention and relaxation technique (n = 3) [32,37,44], prolonged exposure for adolescents (PE-A) based on narrative psychotherapy (n = 1), arts-based pre-texts intervention (n = 1) [39], emotional writing and positive writing (n = 1) [36] and evidence-based family economic empowerment (FEE) and multiple family group (MFG) interventions (n = 1) [41]. The most comparator conditions included were waitlists (n = 8), study skill (n = 4), supportive interventions (n = 1), and control group (n = 1).

More than half (n = 8) of the interventions were delivered by non-specialists such as teachers [36,40], lay providers [31,33,38], per-lead facilitators [39], and community health workers [34,41]. Six (n = 6) interventions were delivered by professionals such as social workers [32,35,37], guidance counselors [43], and psychiatrists [42,44]. Almost, all interventions delivered by non-specialists produced a greater reduction in adolescents’ depressive, anxiety, and post-traumatic stress symptoms compared to the control groups.

The number of intervention sessions among fourteen RCTs ranging from single to 16 sessions. One session length ranged from the shortest 30 minutes to a maximum of 90 minutes. Follow-up durations of interventions across the included studies ranged from one week [37,40,43] to 2 years [34], with the median length of follow-up 12 weeks. Six studies had an intervention follow-up period between 6 months to two years [31,32,34–36,41], and the remaining studies had less than six months of follow up [33,37–40,42–44] (Table 2).

**Table 2:** Psychological interventions characteristics of the included studies (n = 14).

Characteristic	Number	Percent
Year of publication 2010–2015 2016–2022	11 3	78.6 21.4
Study of country Kenya Nigeria South Africa Sierra Leone Mauritius Rwanda Uganda	5 3 2 1 1 1 1	35.71 21.43 14.29 7.14 7.14 7.14 7.14
Mental health condition assessed Depression Anxiety PTSD Depression and anxiety Depression, anxiety and PTSD	6 2 1 3 2	42.86 14.29 7.14 21.42 14.29
Intervention modality Individuals Group	3 11	21.43 78.57
Each session duration 30 minutes 60 minutes 90 minutes	3 8 3	21.43 57.14 21.43
Intervention length < 3 months 1–3 months >3 months	6 5 3	42.86 35.71 21.43
Intervention follow up < 6 months 6–24 months	8 6	57.14 42.86

## Psychological outcomes: Anxiety, depression and post-traumatic stress

Based on pre- and post-intervention assessments, the effectiveness of psychological interventions in reducing the symptoms of depression, anxiety, or PTSD was determined. Post-intervention results showed a significant reduction in symptoms for depression [32,36,38,40–42], anxiety [33], post-traumatic stress symptoms [34,37], and both depression and anxiety [31,39] when compared to control groups. The overall characteristics of the studies are found in Table 3.

**Table 3:** Overall characteristics of the included studies in the review.

Author, year, country	Age range	Sample size	mental health condition	Delivery method	Types of Intervention	Duration of intervention
Rossouw J, et al;2018 South Africa [34]	13–18	64	PTSD	Individual	prolonged exposure for adolescents (PE-A)	14 weeks
Thurman TR, et al;2017, south Africa [32]	13–17	453	Depression	Group	Abangane, psychological group grief intervention	3 months
Betancourt TS,et al; 2014, Sierra Leone [35]	15–24	436	psychological distress, PTSD	Group	Youth Readiness Intervention	6 months
Osborn TL, et al; 2021, Kenya [31]	13–18	413	Depression, anxiety	Group	Shamiri interventions	4 weeks
Getanda EM,et al; 2020, Kenya [37]	14–17	54	Anxiety, depression, PTSD	Individuals	Writing for Recovery (psychosocial educational	3 days
Osborn TL, et al; 2020, Kenya [38]	13–18	103	Depressive, anxiety	Group on computer	Shamiri-Digital intervention	90 minutes
Osborn TL, et al; 2022, Kenya [39]	12–19	235	Depression/ anxiety	Group	Arts-based Pre-Texts intervention	1 week
Venturo-Conerly Etal; 2022, Kenya [33]	14–18	895	Anxiety symptoms	Group	Shamiri intervention (Growth, value, gratitude)	1 day
Jibunoh O,et al; 2022, Nigeria [44]	13–16	40	Anxiety	Group	Group-based psycho-education about anxiety and relaxation techniques	3 weeks
Are A,et al; 2022, Nigeria [40]	13–18	40	depression	Group	manualised group-based CBT	5 weeks
Bella‐Awusah T,et al; 2016, Nigeria [42]	14–17	40	depression	Group	brief school-based CBT	5 weeks
Rivet‐Duval E, et al;2011, Mauritius [36]	12–16	160	depression	Group	The Resourceful Adolescent Program (Adolescent and Parent versions)	11 weeks
Unterhitzenberger J, et al; 2014, Rwanda [43]	14–18	69	grief and depression	Individual	Emotional writing and positive writing	3 weeks
Byansi W, et al; 2022, Uganda [41]	14–17	1260	depressive symptoms	Group	evidence-based family economic empowerment &multiple family group interventions	16 weeks
Author, year, country	follow up length	sessions number	Control group	Therapists	outcomes measures	Main results
Rossouw J, et al;2018 South Africa [34]	24 months	7–14	supportive counselling	Nurses	CPSS-I-24 BDI	Greater improvement on the PTSD symptom severity scale than those receiving supportive counselling (between group differences at post-intervention, mean 12.49, 95% CI 6.82–18.17, P<0.001; d = 1.22). A similar effect size was maintained at 3-month (d = 0.85) and 6-month (d = 1.02) follow-up assessments.
Thurman TR, et al;2017, south Africa [32]	6 months	12	waitlist	social workers	CES–DC-20	the intervention group had significantly lower scores for depression (p = 0·009, d = –0·21) relative to the waitlisted group
Betancourt TS, et al; 2014, Sierra Leone [35]	8 months	10	waitlist	social workers	PD-40, PTSD-12	Not differed significantly in psychological distress and posttraumatic stress symptoms at post-interventions compared to controlled groups
Osborn TL, et al; 2021, Kenya [31]	7 months	4	Study skill	Layperson	GAD-7 PHQ-8	Greater reductions in depressive and anxiety symptoms
Getanda EM, et al; 2020, Kenya [37]	7 days	6	waitlist	social workers	AS-37 DSRC-18	Reduce PTSD, and enhancing quality of life scores but not depressive or anxiety symptoms
Osborn TL, et al; 2020, Kenya [38]	2 weeks	1	Study skill	trained lay-providers	GAD-7 PHQ-8	Shamiri-Digital produced greater reduction in adolescent depression symptoms (p = 0.028, d = 0.50), but no significant effects on anxiety symptoms, well-being, or happiness.
Osborn TL, et al; 2022, Kenya [39]	4 weeks	5	Study skill	Per-led facilitator	GAD-7 PHQ-8	Pre-Texts produced more rapid declines in depression and anxiety from baseline to 1-month follow-up than control-group youths
Venturo-Conerly et al; 2022, Kenya [33]	2 weeks	1	Study skill	trained lay-providers	GAD-7 PHQ-8	greater reductions in anxiety symptoms than the study-skills control (p < .05; d = 0.31 [0.13–0.50])
Jibunoh O,et al; 2022, Nigeria [44]	8 weeks	3	waitlist	psychiatrist	SCAS-38, SMFQ-13	brief psycho-educational intervention for anxiety was not effective in reducing anxiety or depressive symptom
Are A,et al; 2022, Nigeria [40]	1 week	5	waitlist	teachers	BDI-21	the intervention group had significantly lower depressive symptoms scores on the BDI and SMFQ 1 week post intervention with large effect sizes
Bella‐Awusah T,et al; 2016, Nigeria [42]	16 weeks	5	waitlist	psychiatrist	BDI-21 SMFQ-13	the intervention group had significantly lower depressive symptoms scores on the BDI and SMFQ 1 week post intervention with large effect sizes
Rivet‐Duval E, et al;2011, Mauritius [36]	6 months	11	waitlist	teachers	RADS-2 BHS-20	Decreased depressive symptoms were found post-intervention, but not at follow-up. Significant changes in self-esteem and coping skills were seen both post-intervention
Unterhitzenberger J, et al; 2014, Rwanda [43]	1 week	3	Non-writing	guidance counsellor	PGQ-A-36 MINI-KID A-9	Depressive symptoms showed no significant change from pre- to post test in the emotional writing condition, whereas they significantly decreased in the control condition
Byansi W, et al; 2022, Uganda [41]	12 months	16	waitlist	CHWs & peer parents	BDI-21	substantial reduction in depressive symptoms from baseline to 12 months

GAD-7, generalized anxiety disorder- 7 items; PHQ-8, Patient health questionnaire- 8 items; BDI-21, Beck Depression Inventory-21 items; SCAS-38, Spence Children’s Anxiety Scale -38 items; SMFQ-13, Short Mood and Feelings Questionnaire -13 items; RADS-2, The Reynolds Adolescent Depression Scale-2 with 30 items; BHS-20, The Beck Hopelessness Scale- 20 true-false items measuring three major aspects of hopelessness (feelings about the future, loss of motivation, and expectations); CPSS-I-24, Child PTSD Symptom Scale–Interview -24 items; CES–DC-20, Center for Epidemiological Studies–Depression Scale for Children- 20 item; PD-40, Psychological distress (28 items: 16 internalizing and 12 externalizing items); AS-37, Anxiety Scale-37 items; DSRC-18, Depression Self-Rating Scale for Children-18 items; PGQ-A-36, Prolonged Grief Questionnaire for Adolescents- 36 items; MINI-KID A-9, Mini International Neuropsychiatric Interview for Children and Adolescents, Part A- 9 items to diagnosis major depression; PTSD-12, Post-Traumatic Stress Disorder Reaction Index-

## Discussion

Fourteen RCTs were conducted on the effectiveness of school-based psychological interventions after contextual adaptations from seven countries in this region, the majority (71.4%) were from Kenya, Nigeria, and South Africa. The most common psychological interventions were CBT (focuses on behavioral activation, cognitive restructuring, and problem-solving) and Shamiri intervention (cultivates a growth mindset, practices gratitude, and takes the value or virtues). In general, psychological interventions provided in school settings were effective in reducing depressive, anxiety and posttraumatic stress symptoms when compared to the controlled groups based on pre-and post- interventions measures.

There was considerable variation in the school-based psychological interventions, modality, intensity of the session, facilitators expertise, duration of interventions and follow-up period to address the commonest mental health problem of adolescents. In our review, the psychological interventions were delivered by different professionals such as psychiatrists, social workers, guidance counselors, Nurses, and teachers. Interventions delivered by trained non-specialists also found a reduction in depressive, anxiety or traumatic stress symptoms. This might be non-specialists can be easily accessible, cost-effective and reduce stigma for majority of adolescents. A meta-analysis revealed that teacher-delivered interventions were more effective than controls in reducing depressive, anxiety, and posttraumatic stress symptoms in students [45,46]. In school settings, task-sharing mental health services to non-specialists like teachers present a distinct potential for the implementing of mental health interventions [47].

Sessions numbers used range from single session to 16 sessions, while the follow-up period is from one week to two years. This is consistent with a systematic review among adolescents suggesting that school-based psychological interventions were successful in reducing mental health problems [48] and symptom reduction achieved in a low number of sessions (12 or less) in school settings [49]. Furthermore, improved outcomes were associated with interventions that lasted up to 16 weeks, had 45-90-minute-long sessions, and included two or more days of teacher training [45].

In this review, the most frequently utilized intervention was CBT. Studies that utilized CBT in this review, found significant reductions in depressive or traumatic stress symptoms. This is consistent with previous study findings that reported that CBT delivered to young people in schools can reduce symptoms of depression and anxiety [16,50,51]. A systematic review and meta-analysis of LMICs showed that CBT reduced depressive symptoms more effectively than other treatments in adolescents [52]. Additionally, interventions delivered in clinic and school settings may be more successful than those in community settings [16].

In this review, Shamiri (a Swahili word for thrive) was the second most common school-based intervention. The Shamiri intervention teaches youths to cultivate a growth mindset, practice gratitude, and take value-aligned action. Four RCTs in Kenya have shown the intervention effectively lowers youth mental health issues like depression and anxiety while enhancing social interactions, academic performance, school atmosphere, and other functioning outcomes [31,33,37,38]. Even though this intervention is reported effective, we understood similar authors published the four articles and we cannot find elsewhere whether it is effective, acceptable and feasible or not in similar context of LMICs.

The majority of interventions in these reviewed articles used a group-based modality. This might be because group therapy became effective for interpersonal and intrapersonal psychological problems. Group therapy can provide many possible advantages that individual therapy does not: understanding of the universality of the problem, improved communication skills, forming relationships, social skills, improved self-esteem, and increased coping skills [53]. A meta-analytic review of 56 articles on child and adolescent group treatments, found that various forms of groups were more effective than control groups, indicating that 73% of children and adolescents were better than control groups [54]. However, it can be difficulty to safeguard confidentiality in group therapy, may not be suitable for people with shyness or social anxiety disorder, and scheduling challenges for group sessions [55].

The improvement was significantly better than control group and depressive, anxiety, or PTSD symptoms were significantly lower within a month of completing psychological therapy [56]. The difference between psychological interventions and control groups was significant after 6–12 months of interventions. This is supported with previous systematic review and meta-analysis that clinically significant improvement, less deterioration and better recovery rate was found compared to control groups [57]. In addition, the overall response rate in psychotherapies two months after baseline was superior to usual care and waitlist [58].

### Implication

This review can be used as baseline evidence for the Ministry of Health and Education, school leaders, teachers, health professionals, and psychologists when selecting evidence-based interventions to support students’ mental health and wellbeing in Sub-Saharan countries and in LMICs with similar socio-economic status. Psychological interventions in school settings are effective at reducing depression, anxiety, and trauma symptoms. This emphasizes the significance of providing mental health services in educational settings since many students experience a range of mental health problems that may affect their achievement and wellness in terms of their academic, relational, social, and physical well-being. Of note, teacher-delivered interventions in school could be less stigmatized, easily accessible, cost-effective, and affordable for most adolescents to fill the large mental health care gap. This in turn, alleviate the four main barriers of adolescents not seeking and accessing help for their mental health problems identified in the previous systematic review. These include, individual factors (limited mental health knowledge), social factors (perceived social stigma and embarrassment), perceptions of the therapeutic relationship (perceived confidentiality and the ability to trust unknown persons), and systemic and structural barriers (financial costs for mental health services, logistical barriers, and the availability of services) [59]. In addition, delivering school-based psychological intervention for students is one of the promises of broadening access to mental health services with sustainable methods of delivery for those under-detected and under-treated adolescent population in low resource settings. Overall, screening and managing internalized mental health problems in school settings is crucial to facilitate a smooth transition from adolescence to adulthood.

### Limitation

Our reviews showed that school-based psychological interventions significantly reduce depressive, anxiety, or PTSD symptoms among adolescents. However, there were several limitations in this review. First, we advise the generalizability of this finding to SSA in caution since there was the over-representation of two countries, under-representation of others, and no representation for most of the countries in SSA. Second, the intervention delivery format, duration of interventions, sessions intensity, and follow up length varied among the included studies. Therefore, to bring a single conclusion on the above-mentioned variability of interventions could not be drawn. Future reviews better examine how these variabilities of interventions may influence their effectiveness. In addition, a few trials found a low risk of bias and nearly half of them were not registered during the quality assessment.

### Conclusion

This systematic review provides evidence on school-based psychological interventions for managing common adolescent mental health problems such as depression, anxiety, and posttraumatic stress disorders in SSA. The review showed school-based psychological interventions had a stronger impact on symptom reduction. The most commonly used interventions from the included studies were CBT and non-CBT approaches. The range of interventions could be effectively delivered by non-professionals such as teachers, per-lead or community leaders that promote task-shifting of psychological interventions from very scarce mental health specialists in these countries. Particularly, psychological interventions provided in schools by teachers or peer-led facilitators can be more affordable, less stigmatized, and easily accessible for the vast majority of adolescents. This makes the interventions could be more scalable as well as sustainable to address the mental health need of adolescents. This, in turn, narrows the very large mental health treatment gaps in SSA, helps the students improve functioning at school, and reduces the distress of the student, teachers, and parents.

## Supporting information

S1 Checklist PRISMA 2020 Checklist for reporting systematic reviews. (DOCX)

S1 File (DOCX)

S2 File (XLSX)
