# Supplementary material for: Effectiveness of school-based psychological interventions for the treatment of depression, anxiety and post-traumatic stress disorder among adolescents in sub-Saharan Africa: A systematic review of randomized controlled trials
Source: PLoS One. 2023 Nov 20;18(11):e0293988. doi: 10.1371/journal.pone.0293988 (PMC10659195; doi:10.1371/journal.pone.0293988)
Supplement: S1 File — (DOCX) [file pone.0293988.s002.docx]

**Searching databases- Effectiveness of psychological intervention for young adolescents**

| Scopus.com | ( TITLE-ABS-KEY ( depression  OR  "depressive symptoms"  OR  "depressive disorder"  OR  "affective disorder"  OR  "major depression"  OR  "major depressive disorder"  OR  "probable depression"  OR  "anxiety disorders"  OR  "Social Anxiety"  OR  anxiousness  OR  anxious  OR  anxiety  OR  "internalized problems"  OR  "post traumatic"  OR  posttraumatic  OR  "post-traumatic"  OR  ptsd  OR  "psychological distress"  OR  " common mental disorders" )  AND  TITLE-ABS-KEY ( youth  OR  teen*  OR  adolescen*  OR  " young people" )  AND  TITLE-ABS-KEY ( "brief group intervention"  OR  "brief psychosocial intervention"  OR  "psychosocial intervention"  OR  "psychological intervention"  OR  " psychological treatment"  OR  "psychological therapy"  OR  "mental health interventions"  OR  "cognitive behavioral therapy"  OR  "psycho-supportive interventions"  OR "peer education programs"  OR  psychoeducation  OR  "self-help intervention"  OR  "stress management"  OR  " psychological service"  OR  "emotional support"  OR  "interpersonal therapy"  OR  " behavioral intervention"  OR "behavioral activations"  OR  "behavioural activations"  OR  "problem solving"  OR  " group therapy"  OR  "Social skills interventions"  OR  psychotherapy  OR  counseling  OR  mindfulness  OR  "Mindfulness and relaxation" ) )  AND  ( LIMIT-TO ( PUBYEAR ,  2022 )  OR  LIMIT-TO ( PUBYEAR ,  2021 )  OR  LIMIT-TO ( PUBYEAR ,  2020 )  OR  LIMIT-TO ( PUBYEAR ,  2019 )  OR  LIMIT-TO ( PUBYEAR ,  2018 )  OR  LIMIT-TO ( PUBYEAR ,  2017 )  OR  LIMIT-TO ( PUBYEAR ,  2016 )  OR  LIMIT-TO ( PUBYEAR ,  2015 )  OR  LIMIT-TO ( PUBYEAR ,  2014 )  OR  LIMIT-TO ( PUBYEAR ,  2013 )  OR  LIMIT-TO ( PUBYEAR ,  2012 )  OR  LIMIT-TO ( PUBYEAR ,  2011 )  OR  LIMIT-TO ( PUBYEAR ,  2010 ) )  AND  ( LIMIT-TO ( DOCTYPE ,  "ar" ) )  AND  ( LIMIT-TO ( LANGUAGE ,  "English" ) )  AND  ( EXCLUDE ( AFFILCOUNTRY ,  "United States" )  OR  EXCLUDE ( AFFILCOUNTRY ,  "United Kingdom" )  OR  EXCLUDE ( AFFILCOUNTRY ,  "Australia" )  OR  EXCLUDE ( AFFILCOUNTRY ,  "Canada" )  OR  EXCLUDE ( AFFILCOUNTRY ,  "Germany" )  OR  EXCLUDE ( AFFILCOUNTRY ,  "Netherlands" )  OR  EXCLUDE ( AFFILCOUNTRY ,  "China" )  OR  EXCLUDE ( AFFILCOUNTRY ,  "Sweden" )  OR  EXCLUDE ( AFFILCOUNTRY ,  "Italy" )  OR  EXCLUDE ( AFFILCOUNTRY ,  "Spain" )  OR  EXCLUDE ( AFFILCOUNTRY ,  "Norway" )  OR  EXCLUDE ( AFFILCOUNTRY ,  "Switzerland" )  OR  EXCLUDE ( AFFILCOUNTRY ,  "India" )  OR  EXCLUDE ( AFFILCOUNTRY ,  "France" )  OR  EXCLUDE ( AFFILCOUNTRY ,  "Turkey" )  OR  EXCLUDE ( AFFILCOUNTRY ,  "Denmark" )  OR  EXCLUDE ( AFFILCOUNTRY ,  "Brazil" )  OR  EXCLUDE ( AFFILCOUNTRY ,  "Japan" )  OR  EXCLUDE ( AFFILCOUNTRY ,  "Israel" )  OR  EXCLUDE ( AFFILCOUNTRY ,  "New Zealand" )  OR  EXCLUDE ( AFFILCOUNTRY ,  "Iran" )  OR  EXCLUDE ( AFFILCOUNTRY ,  "Belgium" )  OR  EXCLUDE ( AFFILCOUNTRY ,  "South Korea" )  OR  EXCLUDE ( AFFILCOUNTRY ,  "Hong Kong" )  OR  EXCLUDE ( AFFILCOUNTRY ,  "Austria" )  OR  EXCLUDE ( AFFILCOUNTRY ,  "Ireland" )  OR  EXCLUDE ( AFFILCOUNTRY ,  "Finland" )  OR  EXCLUDE ( AFFILCOUNTRY ,  "Malaysia" )  OR  EXCLUDE ( AFFILCOUNTRY ,  "Portugal" )  OR  EXCLUDE ( AFFILCOUNTRY ,  "Pakistan" )  OR  EXCLUDE ( AFFILCOUNTRY ,  "Taiwan" )  OR  EXCLUDE ( AFFILCOUNTRY ,  "Greece" )  OR  EXCLUDE ( AFFILCOUNTRY ,  "Poland" )  OR  EXCLUDE ( AFFILCOUNTRY ,  "Singapore" ) )  AND  ( EXCLUDE ( SUBJAREA ,  "AGRI" )  OR  EXCLUDE ( SUBJAREA ,  "BIOC" )  OR  EXCLUDE ( SUBJAREA ,  "BUSI" )  OR  EXCLUDE ( SUBJAREA ,  "COMP" )  OR  EXCLUDE ( SUBJAREA ,  "DENT" )  OR  EXCLUDE ( SUBJAREA ,  "EART" )  OR  EXCLUDE ( SUBJAREA ,  "ECON" )  OR  EXCLUDE ( SUBJAREA ,  "ENER" )  OR  EXCLUDE ( SUBJAREA ,  "ENGI" ) ) | 422 articles |
| --- | --- | --- |
| Pubmed.gov | Efect* OR efficac* AND (depression[Title/Abstract] OR "depressive symptoms"[Title/Abstract] OR "depressive disorder"[Title/Abstract] OR "affective disorder"[Title/Abstract] OR "major depression"[Title/Abstract] OR "major depressive disorder"[Title/Abstract] OR "probable depression"[Title/Abstract] OR "anxiety disorders"[Title/Abstract] OR "Social Anxiety"[Title/Abstract] OR Anxiousness[Title/Abstract] OR anxious[Title/Abstract] OR anxiety[Title/Abstract] OR "internalized problems"[Title/Abstract] OR "post traumatic"[Title/Abstract] OR posttraumatic[Title/Abstract] OR "post¬traumatic"[Title/Abstract] OR PTSD[Title/Abstract] OR "psychological distress"[Title/Abstract] OR " common mental disorders"[Title/Abstract] AND youth[Title/Abstract] OR teen*[Title/Abstract] OR adolescen*[Title/Abstract] OR " young people"[Title/Abstract] AND "brief group intervention"[Title/Abstract] OR "brief psychosocial intervention"[Title/Abstract] OR "psychosocial intervention"[Title/Abstract] OR "psychological intervention"[Title/Abstract] OR " psychological treatment"[Title/Abstract] OR "psychological therapy"[Title/Abstract] OR "mental health interventions"[Title/Abstract] OR "cognitive behavioral therapy"[Title/Abstract] OR "psycho-supportive interventions"[Title/Abstract] OR "peer education programs"[Title/Abstract] OR Psychoeducation[Title/Abstract] OR "self-help intervention"[Title/Abstract] OR "stress management"[Title/Abstract] OR " psychological service"[Title/Abstract] OR "emotional support"[Title/Abstract] OR "interpersonal therapy"[Title/Abstract] OR " behavioral intervention"[Title/Abstract] OR "behavioral activations"[Title/Abstract] OR "behavioural activations"[Title/Abstract] OR "problem solving"[Title/Abstract] OR " group therapy"[Title/Abstract] OR "Social skills interventions"[Title/Abstract] OR psychotherapy[Title/Abstract] OR counseling[Title/Abstract] OR mindfulness[Title/Abstract] OR "Mindfulness and relaxation"[Title/Abstract]) AND "school-based" OR "class-room" OR "secondary school" OR school AND (Africa South of the Sahara[Title/Abstract] OR Sub Saharan Africa[Title/Abstract] OR sub-Saharan Africa[Title/Abstract] OR Sub-Saharan Africa[Title/Abstract] OR Angola[Title/Abstract] OR Benin[Title/Abstract] OR Botswana[Title/Abstract] OR Burkina Faso[Title/Abstract] OR Upper Volta[Title/Abstract] OR Burundi[Title/Abstract] OR Cameroon[Title/Abstract] OR Cape Verde[Title/Abstract] OR Central African Republic[Title/Abstract] OR Chad[Title/Abstract] OR Comoros[Title/Abstract] OR Congo[Title/Abstract] OR Cote D’ivoire[Title/Abstract] OR Ivory Coast[Title/Abstract] OR Zaire[Title/Abstract] OR Democratic Republic Of The Congo[Title/Abstract] OR French Somaliland[Title/Abstract] OR Djibouti[Title/Abstract] OR Equatorial Guinea[Title/Abstract] OR Eritrea[Title/Abstract] OR Ethiopia[Title/Abstract] OR Gabonese Republic[Title/Abstract] OR Gabon[Title/Abstract] OR Gambia[Title/Abstract] OR Gold Coast[Title/Abstract] OR Ghana[Title/Abstract] OR Guinea[Title/Abstract] OR Guinea-Bissau[Title/Abstract] OR Kenya[Title/Abstract] OR Basutoland[Title/Abstract] OR Lesotho[Title/Abstract] OR Liberia[Title/Abstract] OR Malagasy Republic[Title/Abstract] OR Madagascar[Title/Abstract] OR Nyasaland[Title/Abstract] OR Malawi[Title/Abstract] OR Mali[Title/Abstract] OR Mauritania[Title/Abstract] OR Mauritius[Title/Abstract] OR Mayotte[Title/Abstract] OR Mozambique[Title/Abstract] OR Namibia[Title/Abstract] OR Niger[Title/Abstract] OR Nigeria[Title/Abstract] OR Reunion[Title/Abstract] OR Rwanda[Title/Abstract] OR Ruanda-Urundi[Title/Abstract] OR Sao Tome & Principe[Title/Abstract] OR Sao Tome[Title/Abstract] OR Senegal[Title/Abstract] OR Seychelles[Title/Abstract] OR Sierra Leone[Title/Abstract] OR Somalia[Title/Abstract] OR South Africa[Title/Abstract] OR South Sudan[Title/Abstract] OR Sudan[Title/Abstract] OR Swaziland[Title/Abstract] OR Eswatini[Title/Abstract] OR Togolese Republic[Title/Abstract] OR Togo[Title/Abstract] OR Uganda[Title/Abstract] OR United Republic Of Tanzania[Title/Abstract] OR Tanzania[Title/Abstract] OR Zambia[Title/Abstract] OR Zimbabwe[Title/Abstract] OR Rhodesia[Title/Abstract] OR Africa Eastern[Title/Abstract] OR Africa Southern[Title/Abstract]) AND ((randomizedcontrolledtrial[Filter]) AND (2010/1/1:2022/12/30[pdat]) AND (child[Filter] OR youngadult[Filter])) AND ((randomizedcontrolledtrial[Filter]) AND (2010/1/1:2022/12/30[pdat]) AND (child[Filter] OR youngadult[Filter])) AND ((randomizedcontrolledtrial[Filter]) AND (2010/1/1:2022/12/30[pdat]) AND (adolescent[Filter] OR child[Filter] OR youngadult[Filter])) | 781 articles |
| Embase | (((depression OR 'depressive symptoms' OR 'depressive disorder' OR 'affective disorder' OR 'major depression' OR 'major depressive disorder' OR 'probable depression' OR 'anxiety disorders' OR 'social anxiety' OR anxiousness OR anxious OR anxiety OR 'internalized problems' OR 'post traumatic' OR posttraumatic OR 'post¬traumatic' OR ptsd OR 'psychological distress' OR 'common mental disorders') AND youth OR teen* OR adolescen* OR 'young people') AND 'brief group intervention' OR 'brief psychosocial intervention' OR 'psychosocial intervention' OR 'psychological intervention' OR 'psychological treatment' OR 'psychological therapy' OR 'mental health interventions' OR 'cognitive behavioral therapy' OR 'psycho-supportive interventions' OR 'peer education programs' OR psychoeducation OR 'self-help intervention' OR 'stress management' OR 'psychological service' OR 'emotional support' OR 'interpersonal therapy' OR 'behavioral intervention' OR 'behavioral activations' OR 'behavioural activations' OR 'problem solving' OR 'group therapy' OR 'social skills interventions' OR psychotherapy OR counseling OR mindfulness OR 'mindfulness and relaxation') AND 'school-based' OR 'class-room' AND (2010:py OR 2011:py OR 2012:py OR 2013:py OR 2014:py OR 2015:py OR 2016:py OR 2017:py OR 2018:py OR 2019:py OR 2020:py OR 2021:py OR 2022:py) AND ([adolescent]/lim OR [school]/lim OR [young adult]/lim) AND 'article'/it | 609 articles found (2010-2022) |
| ScienceDirect | "school based" AND adolescents AND "depressive symptoms OR "post-traumatic stress disorders" AND "psychological interventions" AND "sub-Saharan Africa" AND "Randomized controlled trials" | 1199 articles |
